# Chitosan Films Incorporated with Exopolysaccharides from Deep Seawater *Alteromonas* sp.

**DOI:** 10.3390/md18090447

**Published:** 2020-08-27

**Authors:** Iratxe Zarandona, Mónica Estupiñán, Carla Pérez, Laura Alonso-Sáez, Pedro Guerrero, Koro de la Caba

**Affiliations:** 1 BIOMAT research group, University of the Basque Country (UPV/EHU), Escuela de Ingeniería de Gipuzkoa, Plaza de Europa 1, 20018 Donostia-San Sebastián, Spain; iratxe.zarandona@ehu.eus; 2 AZTI Marine Research, Basque Research and Technology Alliance (BRTA), Txatxarramendi ugartea z/g, 48395 Sukarrieta, Spain; mestupinan@azti.es (M.E.); carlaperez@azti.es (C.P.); lalonso@azti.es (L.A.-S.)

**Keywords:** chitosan, exopolysaccharides, *Alteromonas*, marine bacteria, films

## Abstract

Two *Alteromonas* sp. strains isolated from deep seawater were grown to promote the production of exopolysaccharides (EPS, E611 and E805), which were incorporated into chitosan solutions to develop films. The combination of the major marine polysaccharides (chitosan and the isolated bacterial EPS) resulted in the formation of homogenous, transparent, colorless films, suggesting good compatibility between the two components of the film-forming formulation. With regards to optical properties, the films showed low values of gloss, in the range of 5–10 GU, indicating the formation of non-glossy and rough surfaces. In addition to the film surface, both showed hydrophobic character, with water contact angles higher than 100 º, regardless of EPS addition. Among the two EPS under analysis, chitosan films with E805 showed better mechanical performance, leading to resistant, flexible, easy to handle films.

## 1. Introduction

Polysaccharides are widely used to develop films and coatings for food and biomedical applications [1] as an alternative to plastic-based film production. In particular, chitosan is a polysaccharide extracted from marine crustacean shells [2], and due to its non-toxicity, antimicrobial activity, and biocompatibility [3], it is suitable as a food preservative [4], drug delivery system [5], and wound healing [6] application. Recently, research has been conducted in order to find novel functionalities of chitosan films through the incorporation of bioactive compounds of natural origin. In particular, the addition of essential oils from two aromatic herbs into chitosan films enhanced their antioxidant and antibacterial activity [7]. Snail mucus extract, a substance that is widely used in cosmetics formulations for its protective and reparative activities, has also been incorporated into chitosan-based films to modulate their properties [8]. Recently, chitosan films have shown their biocompatibility and wound healing capacity [9], suggesting their potential to develop more sophisticated wound dressings based on chitosan, including other bioactive molecules.

In addition to other marine sources, like animals, seaweeds, and invertebrates, microorganisms provide glycopolymers that display great diversity in structures and composition. These original chemical structures are frequently linked to promising biological activities [10] and thus represent a target for biodiscovery. In general, EPS are high molecular weight polymers constituted of homopolysaccharides or heteropolysaccharides, which can form linear or branched structures [11,12]. EPS derived from marine bacteria are currently attracting substantial attention [13] as they show interesting properties. These EPS include atypical sugar monomers, such as fructose and rhamnose [14], which are of commercial interest, and they typically contain several organic and inorganic substitutes that modulate their physicochemical properties. For instance, the adhesive and viscous properties of EPS are attributed to the presence of uronic acids, sulphates, or carboxyl groups that confer anionic properties to the polymer [15].

EPS are used as thickening, gelling, stabilizing, or emulsifying agents [16] and they have also been exploited as a source of new biomaterials. Since EPS are biocompatible, biodegradable, and have good adhesion capacity to cells [17,18], they are increasingly used in the fields of biotechnology and biomedicine, despite their cost of production being high for most commercial applications. As an example of biomedical applications, the EPS isolated from *Pantoea* sp. have been assessed for cutaneous wound healing and tissue engineering [19], while nanoparticles for drug delivery were obtained after complexing the anionic EPS secreted from *Lactobacillus acidophilus* and *Halomonas maura* with chitosan, a positively charged polymer [20].

The genus *Alteromonas* is ubiquitously found in marine environments, from surface coastal seawater to the deep ocean [21], and it represents a promising source of a wide range of metabolites including EPS, antimicrobial, and antitumoral agents. Members of this genus have a great diversity of genes involved in the synthesis of EPS [22], likely essential for cell–cell interaction and recognition, biofilm formation, or nutrient uptake. It has been found that *Alteromonas* HYD-1545 secretes an EPS with high levels of uronic acids and pyruvate, showing anticoagulant and bone healing properties [23]. Moreover, the EPS from *Alteromonas macleodii* subsp. *fijiensis* is currently commercialized for cosmetic purposes under the name Abyssine^®^, and it is able to reduce skin irritation against chemical, mechanical, and UVB (ultraviolet B) aggression. Additionally, *Alteromonas* strain 1644 secretes an EPS that is capable of binding heavy metals [24,25].

The aim of this work is to analyze the compatibility between the EPS produced by *Alteromonas* strains isolated from deep (≥500 m depth) seawaters, and a polysaccharide like chitosan in order to develop sustainable films from marine-derived biopolymers. In particular, in a previous work, some of the bacteria selected for the EPS-screening assay were found to produce omega-3 [26], and a further valorization of these microorganisms, together with the assessment of their compatibility with chitosan to develop films, are the main aims of the current study. Hence, EPS were incorporated into chitosan formulations, resulting in homogenous films, indicating good compatibility between these two biopolymers derived from marine sources. Functional properties of these films, such as physicochemical, optical, barrier, and mechanical properties, were assessed and related to the film structure.

## 2. Results and Discussion

### 2.1. Screening of Marine Bacterial Exopolysaccharides

A collection of 95 gamma-proteobacteria from *Pseudoalteromonas* (51), *Alteromonas* (15), and *Vibrio* (19) genus marine isolates were grown in the absence or presence of sucrose as the major carbon source. Highly sucrose-induced mucoid colony phenotypes were mostly observed in *Alteromonas* sp. deep sea water isolates (66.7%), compared to *Pseudoalteromonas* and *Vibrio* sp. isolates (17.7% and 15.8%, respectively). None of the EPS-producing bacteria showed the characteristic black phenotype in CR agar, suggesting the production of novel EPS variants in marine isolates. In a previous work, phylogenetic studies based on partial sequence of 16 S rDNA of all tested deep seawater *Alteromonas* sp. showed a high percentage of identity (≥99%) [26]. However, the isolates showed phenotypic variation, as three different colony morphologies were observed in sucrose-induced plates. *Alteromonas* sp. 805 and 611 were selected as representatives of two of the colony morphologies found.

### 2.2. Production of EPS

In the batch experiment, an EPS production of 1.0 g/L (E805) and 0.7 g/L (E611) was observed after 96 h of culture at 10 °C in non-optimized conditions, as typically found for marine EPS-producing strains (i.e., 0.5 to 4.0 g/L of EPS in the presence of glucose) [27]. Moreover, the crude EPS contained 0.1% of DNA and 2% of protein in both strains.

### 2.3. Optical Properties

**Figure 1:**
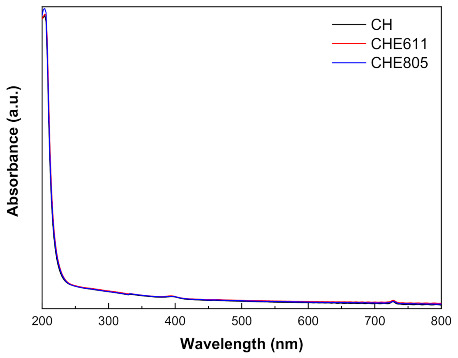
UV–vis light absorption of control (CH) and chitosan films with E805 (CHE805) and E601 (CHE601).

The effect of EPS supplementation on chitosan film appearance was determined by UV–vis absorbance, color, and gloss measurements. In general, chitosan films were transparent and colorless with a subtle yellow tinge. As can be seen in Figure 1, there was no light absorbance in the visible range from 400 to 800 nm, indicating that control films and those with EPS were transparent. Additionally, all films absorbed UV light at 200 nm, corresponding to the carbonyl groups in chitosan [28].

Color changes were analyzed by determining L *, a *, and b * parameters, and results are shown in Table 1. E811 did not significantly (*p* > 0.05) affect the color parameters of chitosan films. However, the addition of E611 caused a significant increase (*p* < 0.05) of b * values and a * values, which became more negative (*p* < 0.05), indicating an increase of yellowness and greenness, respectively. L * values did not decrease (*p* > 0.05), indicating that CHE611 films showed high lightness. It is worth noting that these changes were not perceptible to the human eye, since ∆E * values were lower than 5 [29]; in particular, values were lower than 1. Therefore, it can be said that the appearance of control chitosan films prevailed after the addition of EPS. Additionally, gloss values were measured and are shown in Table 1. All films showed very low gloss values, indicating that the film surface was very rough, since low gloss values are related to high surface roughness [30]. Moreover, chitosan films supplemented with E805 were found to be even rougher since gloss values decreased from 10 to 5 GU.

### 2.4. Barrier and Mechanical Properties

Following the analysis of film surface, the hydrophilic or hydrophobic character of chitosan films was assessed by measuring water contact angle (WCA) values. As shown in Table 2, control chitosan films were hydrophobic since the WCA values were greater than 90° [31]. No significant (*p* > 0.05) difference was found for CHE805 films, while a significant (*p* < 0.05) increase was observed for CHE611 films with values of 115°. These results are in accordance with the abovementioned gloss values, indicating a rougher surface for CHE611 films.

In relation to water-related properties, there was no significant (*p* > 0.05) difference in WVP values with the addition of EPS (Table 2). Permeability values depended on adsorption, diffusion, and desorption processes. Although CHE611 films were more hydrophobic, similar values of WVP would indicate that the diffusion process is more rapid in these films.

Tensile tests were performed in order to evaluate the effect of EPS addition on tensile strength (TS), elongation at break (EAB), and elastic modulus (EM). The addition of E611 caused a significant (*p* < 0.05) decrease in TS, EAB, and EM values, but E805 did not significantly (*p* > 0.05) change the mechanical performance of chitosan films (Table 2), resulting in resistant and flexible films which were easy to handle. This behavior is in accordance with previous analyses, which also led to similar results for control and CHE805 films, while CHE611 films showed changes in both optical and barrier properties compared to control and CHE805 films.

### 2.5. Physicochemical Properties and Film Morphology

**Figure 2:**
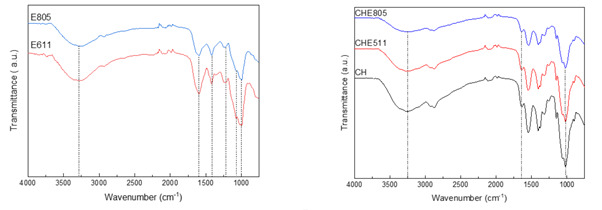
FTIR (Fourier Transform Infrared) spectra of (**a**) E611 and E805 and (**b**) those of control (CH) and chitosan films with E611 (CHE611) and E805 (CHE805).

SpainFTIR (Fourier Transform Infrared) analysis was carried out to evaluate the interactions between chitosan and EPS. Regarding EPS (Figure 2a), the main characteristic bands appeared at 3300 cm^−1^, corresponding to O-H stretching; at 1633 cm^−1^, associated to C=O stretching; around 1400 cm^−1^, corresponding to C-H stretching vibrations in hexoses; at 1230 cm^−1^, associated to the presence of sulfates; at 1100 cm^−1^, due to glycosidic linkages; and at 1000 cm^−1^, related to C-O stretching [14]. The most relevant difference between the two EPS was related to the difference in the relative intensity between the bands at 1633 and 1400 cm^−1^. As can be seen in Figure 2a, the intensity of those bands was similar for E805, indicating a higher content of hexoses, which could lead to a higher TS of CHE805 films due to these cyclic structures providing rigidity. Additionally, a higher EAB of CHE805 films could be due to the promotion of hydrogen bonding with the hydroxyl groups of the hexoses.

When EPS were incorporated into chitosan film forming formulations, some shifts of the abovementioned characteristic bands were observed, as shown in Figure 2b. In particular, the band at 3300 cm^−1^ was shifted to 3250 cm^−1^, the band at 1633 cm^−1^ to 1655 cm^−1^, and the band at 1000 cm^−1^ to 1030 cm^−1^ for CHE805 and CHE611 films. These shifts are indicative of physical interactions among chitosan, glycerol, and EPS, mainly by hydrogen bonding among the polar groups (hydroxyl and carbonyl groups) of the components of the film forming formulation.

**Figure 3:**
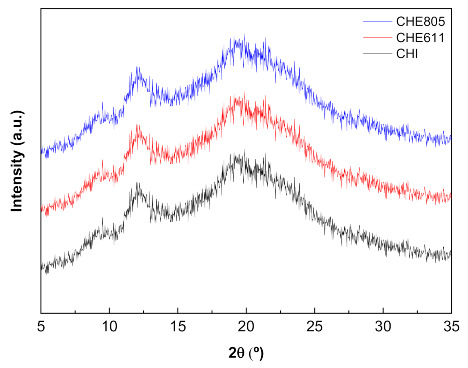
XRD (X-ray diffraction) patterns of control (CH) and chitosan films with E611 (CHE611) and E805 (CHE805).

Finally, XRD (X-ray diffraction) and SEM (Scanning Electron Microscopy) analyses were carried out in order to assess the film structure. The three characteristic peaks of chitosan were observed at 9°, 12°, and 20° [8] for all the chitosan films under study, regardless of the addition of EPS (Figure 3).

**Figure 4:**
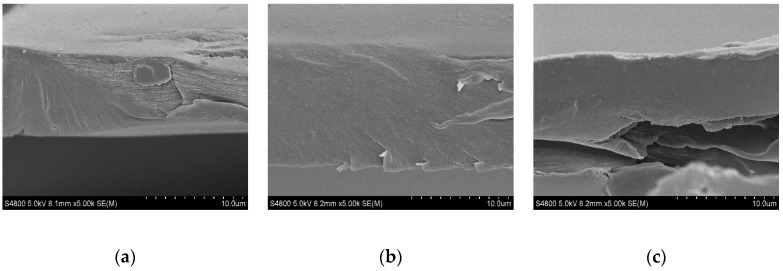
SEM (Scanning Electron Microscopy) images of cross-sections of (**a**) control films and chitosan films enriched with (**b**) E611 and (**c**) E805 exopolysaccharides.

Regarding SEM analysis, film cross-sections are shown in Figure 4. A compact structure was observed for all films, suggesting good compatibility among the film components.

## 3. Materials and Methods

### 3.1. Materials

Chitosan, with a molecular weight of 375 kDa and deacetylation degree above 75%, was supplied by Sigma-Aldrich, Spain. Acetic acid solution (1 N) and glycerol (99.0% purity), used as solvent and a plasticizer, respectively, were supplied by Panreac, Spain.

### 3.2. Screening of EPS-Producing Marine Bacteria

To select exopolysaccharide (EPS) bacterial producers, a screening of free-living Gram-negative bacteria (95) belonging to *Pseudoalteromonas*, *Alteromonas*, and *Vibrio* sp. strains, isolated from different depths in the same seawater column (5, 500 and 1000 m depth) [26], was performed in agar plates (25 °C, 6 d). Marine Artificial Seawater (MASW) plates (38 g of Instant Ocean^®^ sea salt, 5 g peptone, and 1 g yeast extract, 15 g agar per liter) were prepared, adding 0.8 g/L of Congo red (CR) [32]. CR dye was used as a colorimetric screening method for the detection of glucan polysaccharides as it binds specifically to β-glucans (β-1,3-d-glucans and β-1,4-d-glucans) [33]. In parallel, a set of CR agar plates were supplemented with 3% sucrose (Fisher Reagents) to induce EPS production. Colony morphology and phenotype of each isolate was compared in both control and carbon-supplemented CRA to observe possible EPS-producing candidates.

### 3.3. Bacterial Strains and Growth Conditions

Two *Alteromonas* strains (611 and 805) isolated from deep seawater samples (1000 m and 500 m depth, respectively) collected in the Bay of Biscay [26] were grown in MASW. For EPS production and isolation, 1 L bottles containing 600 mL of MASW were inoculated at 2% (*v/v*) from a starter overnight culture incubated at 25 °C, 190 rpm. To induce EPS production, *Alteromonas* sp. 611 culture media MASW was supplemented with an additional carbon source (3% of sucrose, Fisher BioReagents) and shaken for 6 d (190 rpm) at 10 °C in aerobic conditions.

### 3.4. EPS Isolation from Alteromonas sp. Isolates

The crude EPS from the bacterial isolates was obtained as previously described [34]. In brief, cells were removed from the medium by centrifugation at 15,000× *g* for 20 min. The supernatant was centrifuged twice in order to reduce the presence of bacteria. EPS was precipitated from the clarified supernatant by addition (1:1, *v/v*) of cold absolute ethanol (−20 °C) (Fisher Scientific), and incubated at 4 °C overnight. The precipitate was harvested by centrifugation (12,000× *g*, 30 min) and washed three times with increasing ratios of ethanol to water (50, 70, 100%). Then, the precipitate was resuspended in deionized water, rehydrated overnight at 4 °C, and freeze-dried. The crude EPS (E611 and E805) were stored at room temperature.

### 3.5. EPS Isolation from Alteromonas sp. Isolates

The yield of EPS production was characterized by gravimetry of the lyophilized crude EPS. Protein and DNA content was measured by Qubit^®^ 2.0 fluorometer (Thermo Fisher Scientific, Waltham, MA, USA).

### 3.6. Film Preparation

Chitosan (1% *w/v*) was dissolved in 1% wt acetic acid solution by mechanical agitation for 45 min at room temperature. EPS (5 wt.%, referred to chitosan dry mass) was added to the chitosan solution, and stirring was maintained for 30 min. In order to homogenize the mixture, Ultraturrax (IKA, Germany) was used at 15,000 rpm for 90 s. Subsequently, 15 wt.% glycerol (chitosan based) was added, and stirring was maintained for 2 h. Solutions were poured into Petri dishes and left to dry at room temperature. The resulting films were named CH for control chitosan films, CHE611 for chitosan films supplemented with E611, and CHE805 for chitosan films with E805.

### 3.7. UV–Vis Spectroscopy

A UV-VIS-NIR Shimadzu spectrometer (Shimadzu Scientific Instruments, Kyoto, Japan) was employed to measure light transmission through the film. The absorbance range was set from 200 to 800 nm.

### 3.8. Color Measurements

Color measurements were collected with a CR-400 Minolta Chroma Meter colorimeter (Konica Minolta, Tokyo, Japan). CIELAB scale was used for color parameter determination: L * from 0 to 100 (from black to white), a * from − to + (from greenness to redness), and b * from − to + (from blueness to yellowness). Measurements were carried out on a standard white plate, and ten replicates were collected for each sample.

### 3.9. Gloss Measurements

Gloss values were determined by a Multi Gloss 268 Plus (Konica Minolta, Tokyo, Japan) with an incidence angle of 60°, according to ASTM (American Society for Testing and Materials) D523-18 [35]. Ten samples were collected for each sample at room temperature.

### 3.10. Water Contact Angle (WCA)

A Dataphysic Contact Angle System, Oca 20 model, was used for WCA measurements. A 3 µL volume of distilled water was dropped onto the film surface, and the drop image was collected using a SCA20 software. Measurements were carried out in quintuplicate.

### 3.11. Water Vapor Permeability (WVP)

PERME™W3/0120 chamber (Labthink Instruments Co. LTD., Shandong, China) was used to measure WVP at a temperature and relative humidity of 38 °C and 90%, respectively, according to ASTM E96-00 [36]. Films were cut with a disc shape of 7.40 cm diameter and a test area of 33 cm^2^. Tests were carried out in triplicate.

### 3.12. Mechanical Properties

An Instron 5967 electromechanical testing system (Instron, MA, USA) was employed for tensile tests, and tensile strength (TS) and elongation at break (EAB) were measured. Tests were carried out with a tensile load of 500 N and a crosshead rate of 5 mm/min, according to ASTM D1708-13 [37]. Samples were cut into a dog bone shape of 4.75 × 22.25 mm. At least five samples for each composition were tested.

### 3.13. Fourier Transform Infrared (FTIR) Spectroscopy

FTIR spectra were collected with a Nicolet Nexus FTIR spectrometer (Thermo Fisher Scientific, Massachusetts, USA) with a Golden Gate ATR accessory. The spectra, with a resolution of 4 cm^−1^, were acquired between 4000 and 800 cm^−1^ with 32 scans for each sample.

### 3.14. X-Ray Diffraction (XRD)

X-ray diffraction (XRD) was carried out at 40 kV and 40 mA, with Cu-Kα (λ = 1.5418 Å) as a radiation source, using a PANalytic Xpert Pro (PANalytical, Almelo, The Netherlands) equipment with a diffraction unit. Data were collected between 2° and 34° (step size = 0.026, time per step = 118 s).

### 3.15. Scanning Electron Microscopy (SEM)

A Hitachi S-4800 scanning electron microscope (Hitachi High-Technologies Corporation, Tokyo, Japan), with an acceleration voltage of 15 kV, was employed to analyze the film cross-sections. Films were placed on a metallic stub and were covered with gold under vacuum in argon atmosphere.

### 3.16. Statistical Analysis

In order to determine significant differences between measurements, analysis of variance (ANOVA) was carried out with SPSS software (SPSS Statistic 24.0.0.2). Tukey’s test with statistical significance at the *p* < 0.05 level was used for multiple comparisons among different systems.

## 4. Conclusions

In this work, screening, isolation, and a preliminary characterization of crude EPS (E611 and E805) produced by two deep seawater *Alteromonas* sp. strains were carried out. All the films obtained were colorless, transparent, and homogeneous. The addition of crude EPS caused an increase of the hydrophobic character of chitosan films, especially for CHE611 films, as shown by higher WCA values. However, WVP prevailed unchanged, indicating that water vapor diffusion was more rapid for CHE611. On the other hand, the addition of E805 led to a better mechanical performance compared to CHE611 films, leading to resistant and flexible films. This preliminary work indicates good compatibility between these two marine polysaccharides from renewable resources and, thus, their potential to develop improved films. Results suggest that the valorization of natural materials from marine sources may be of great interest for the development of potentially active films for biomedical applications, offering an alternative and advanced perspective to increase therapeutic resources for a wide variety of applications. In this regard, in vitro and in vivo assays should be conducted to verify the biocompatibility and capacity of these films to promote cell adhesion and proliferation.

## Figures and Tables

**Table 1:** L *, a *, b *, and ∆E * color parameters and gloss values of control (CH) and chitosan films with E805 (CHE805) and E601 (CHE601).

Films	L*	a*	b*	∆E *	Gloss_60_ (GU)
CH	95.5 ± 0.5 ^a^	−0.08 ± 0.03 ^a^	2.39 ± 0.06 ^a^	---	10 ± 2 ^a^
CHE805	96.8 ± 0.3 ^a^	−0.12 ± 0.03 ^a^	2.56 ± 0.09 ^a^	0.67	10 ± 2 ^a^
CHE611	96.6 ± 0.5 ^a^	−0.22 ± 0.09 ^b^	3.04 ± 0.40 ^b^	0.33	5 ± 2 ^b^

^a,b^ Two means followed by the same letter in the same column are not significantly (*p* > 0.05) different through the Tukey’s multiple range test.

**Table 2:** Water contact angle (WCA), water vapor permeability (WVP), tensile strength (TS), elongation at break (EAB), and elastic modulus (EM) of control (CH) and chitosan films with E805 (CHE805) and E611 (CHE611).

Films	WCA (°)	WVP·10^9^ (g/cm·s·Pa)	TS (MPa)	EAB (%)	EM (MPa)
CH	105 ± 2 ^a^	1.37 ± 0.07 ^a^	41.6 ± 1.0 ^a^	24.7 ± 2.1 ^a^	1193 ± 24 ^a^
CHE805	109 ± 1 ^a^	1.51 ± 0.02 ^a^	42.7 ± 1.5 ^a^	23.7 ± 1.9 ^a^	1186 ± 29 ^a^
CHE611	115 ± 3 ^b^	1.54 ± 0.01 ^a^	39.5 ± 0.5 ^b^	16.6 ± 1.3 ^b^	1008 ± 26 ^b^

^a,b^ Two means followed by the same letter in the same column are not significantly (*p* > 0.05) different through the Tukey’s multiple range test.

## References

[1] Ghasemlou M., Khodaiyan F., Oromiehie A., Yarmand M.S. (2011). Development and characterisation of a new biodegradable edible film made from kefiran, an exopolysaccharide obtained from kefir grains. Food Chem..

[2] Negm N.A., Hefni H.H.H., Abd-Elaal A.A.A., Badr E.A., Kana M.T.H.A. (2020). Advancement on modification of chitosan biopolymer and its potential applications. Int. J. Biol. Macromol..

[3] Riezk A., Raynes J.G., Yardley V., Murdan S., Croft S.L. (2020). Activity of chitosan and its derivatives against *Leishmania major* and *Leishmania mexicana* in vitro. Antimicrob. Agents Chemother..

[4] Hu Z., Gänzle M.G. (2018). Challenges and opportunities related to the use of chitosan as a food preservative. J. Appl. Microbiol..

[5] Ways T.M.M., Lau W.M., Khutoryanskiy V.V. (2018). Chitosan and its derivatives for application in mucoadhesive drug delivery systems. Polymers.

[6] Andonegi M., Las Heras K., Santos-Vizcaíno E., Igartua M., Hernandez R.M., de la Caba K., Guerrero P. (2020). Structure-properties relationship of chitosan/collagen films with potential for biomedical applications. Carbohydr. Polym..

[7] Ruiz-Navajas Y., Viuda-Martos M., Sendra E., Perez-Alvarez J.A., Fernández-López J. (2013). In vitro antibacterial and antioxidant properties of chitosan edible films incorporated with *Thymus moroderi* or *Thymus piperella* essential oils. Food Control.

[8] Di Filippo M.F., Panzavolta S., Albertini B., Bonvicini F., Gentilomi G.A., Orlacchio R.R., Passerini N., Bigi A., Dolci L.S. (2020). Functional properties of chitosan films modified by snail mucus extract. Int. J. Biol. Macromol..

[9] Garcia-Orue I., Santos-Vizcaino E., Etxabide A., Uranga J., Bayat A., Guerrero P., Igartua M., de la Caba K., Hernandez R.M. (2019). Development of bioinspired gelatin and gelatin/chitosan bilayer hydrofilms for wound healing. Pharmaceutics.

[10] Delbarre-Ladrat C., Sinquin C., Lebellenger L., Zykwinska A., Colliec-Jouault S. (2014). Exopolysaccharides produced by marine bacteria and their applications as glycosaminoglycan-like molecules. Front. Chem..

[11] Mohamed S.S., Amer S.K., Selim M.S., Rifaat H.M. (2018). Characterization and applications of exopolysaccharide produced by marine *Bacillus altitudinis* MSH2014 from Ras Mohamed, Sinai, Egypt. Egypt J. Basic Appl. Sci..

[12] Zhao D., Jiang J., Du R., Guo S., Ping W., Ling H., Ge J. (2019). Purification and characterization of an exopolysaccharide from *Leuconostoc lactis* L2. Int. J. Biol. Macromol..

[13] Selim M.S., Amer S.K., Mohamed S.S., Mounier M.M., Rifaat H.M. (2018). Production and characterisation of exopolysaccharide from *Streptomyces carpaticus* isolated from marine sediments in Egypt and its effect on breast and colon cell lines. J. Genet. Eng. Biotechnol..

[14] Sahana T.G., Rekha P.D. (2019). A bioactive exopolysaccharide from marine bacteria *Alteromonas sp.* PRIM-28 and its role in cell proliferation and wound healing in vitro. Int. J. Biol. Macromol..

[15] Wang Y., Compaoré-Sérémé D., Sawadogo-Lingani H., Coda R., Katina K., Maina N.H. (2019). Influence of dextran synthesized in situ on the rheological, technological and nutritional properties of whole grain pearl millet bread. Food Chem..

[16] De Oliveira J.M., Amaral S.A., Burkert C.A.V. (2018). Rheological, textural and emulsifying properties of an exopolysaccharide produced by *Mesorhizobium loti* grown on a crude glycerol-based medium. Int. J. Biol. Macromol..

[17] Ale E.C., Rojas M.F., Reinheimer J.A., Binetti A.G. (2020). *Lactobacillus fermentum*: Could EPS production ability be responsible for functional properties?. Food Microbiol..

[18] Tabernero A., Cardea S. (2020). Supercritical carbon dioxide techniques for processing microbial exopolysaccharides used in biomedical applications. Mater. Sci. Eng. C.

[19] Sahana T.G., Rekha P.D. (2020). A novel exopolysaccharide from marine bacterium *Pantoea* sp. YU16-S3 accelerates cutaneous wound healing through Wnt/β-catenin pathway. Carbohydr. Polym..

[20] Wang J., Salem D.R., Sani R.K. (2019). Extremophilic exopolysaccharides: A review and new perspectives on engineering strategies and applications. Carbohydr. Polym..

[21] García-Martínez J., Acinas S.G., Massana R., Rodriguez-Valera F. (2002). Prevalence and microdiversity of *Alteromonas macleodii*-like microorganisms in different oceanic regions. Environ. Microbiol..

[22] López-Pérez M., Rodriguez-Valera F. (2016). Pangenome evolution in the marine bacerium *Alteromonas*. Genome Biol. Evol..

[23] Vincent P., Pignet P., Talmont F., Bozzi L., Fournet B., Guezennec J., Jeanthon C., Prieur D. (1994). Production and characterization of an exopolysaccharide excreted by a deep-sea *Alvinella pompejana*. Appl. Environ. Microbiol..

[24] Le Costaouëc T., Cérantola S., Ropartz D., Ratiskol J., Sinquin C., Colliec-Jouault S., Boisset C. (2012). Structural data on a bacterial exopolysaccharide produced by a deep-sea *Alteromonas macleodii* strain. Carbohydr. Polym..

[25] Finore I., Di Donato P., Mastascusa V., Nicolaus B., Poli A. (2014). Fermentation technologies for the optimization of marine microbial exopolysaccharide production. Mar. Drugs.

[26] Estupiñán M., Hernández I., Saitua E., Bilbao M.E., Mendibil I., Ferrer J., Alonso-Sáez L. (2020). Novel *Vibrio* spp. strains producing omega-3 fatty acids isolated from coastal seawater. Mar. Drugs.

[27] Roca C., Lehmann M., Torres C.A.V., Baptista S., Gaudêncio S.P., Freitas F., Reis M.A.M. (2016). Exopolysaccharide production by a marine *Pseudoalteromonas* sp. strain isolated from Madeira Archipelago ocean sediments. New Biotechnol..

[28] Ji F., You L., Wang L., Liu Z., Zhang Y., Lv S. (2016). Layer-by-layer assembled chitosan-based antibacterial films with improved stability under alkaline conditions. Ind. Eng. Chem. Res..

[29] Luchese C.L., Abdalla V.F., Spada J.C., Tessaro I.C. (2018). Evaluation of blueberry residue incorporated cassava starch film as pH indicator in different simulants and foodstuffs. Food Hydrocoll..

[30] Sanchez-Gonzalez L., Chafer M., Chiralt A., Gonzalez-Martinez C. (2010). Physical properties of edible chitosan films containing bergamot essential oil and their inhibitory action on *Penicillium italicum*. Carbohydr. Polym..

[31] Grande-Tovar C.D., Serio A., Delgado-Ospina J., Paparella A., Rossi C., Chaves-López C. (2018). Chitosan films incorporated with *Thymus capitatus* essential oil: Mechanical properties and antimicrobial activity against degradative bacterial species isolated from tuna (*Thunnus* sp.) and swordfish (*Xiphias gladius*). J. Food Sci. Technol..

[32] Freeman D.J., Falkiner F.R., Keane C.T. (1989). New method for detecting slime production by coagulase negative staphylococci. J. Clin. Pathol..

[33] Wood P., Fulcher R. (1978). Interaction of some dyes with cereal β-glucans. Cereal Chem..

[34] Rougeaux H., Pichon R., Kervarec N., Raguénès G.H.C., Guezennec J.G. (1996). Novel bacterial exopolysaccharides from deep-sea hydrothermal vents. Carbohydr. Polym..

[35] American Society for Testing and Materials (ASTM) (2018). Standard test method for specular gloss, D523-18. Annual Book of ASTM Standards.

[36] American Society for Testing and Materials (ASTM) (2000). Standard test methods for water vapor transmission of materials, E96-00. Annual Book of ASTM Standards.

[37] American Society for Testing and Materials (ASTM) (2013). Standard test method for tensile properties of plastics, D1708-13. Annual Book of ASTM Standards.

